# Human versus AI in audiological practice: A comparative evaluation of ChatGPT and physician treatment decisions in idiopathic sudden sensorineural hearing loss

**DOI:** 10.1371/journal.pone.0350549

**Published:** 2026-06-01

**Authors:** Valeria Caragli, Michele Pellegrino, Luca Pingani, Gian Maria Galeazzi, Davide Soloperto, Elisabetta Genovese, Cosimo de Filippis, Gino Marioni, Leonardo Franz

**Affiliations:** 1 Audiology Program, Otorhinolaryngology Unit, Department of Medical and Surgical Sciences for Children and Adults, University of Modena and Reggio Emilia, Modena, Italy; 2 Department of Biomedical, Metabolic and Neural Sciences, University of Modena and Reggio Emilia, Modena, Italy; 3 Department of Integrated Mental Health and Substance Abuse Services, Azienda USL - IRCCS of Reggio Emilia, Reggio Emilia, Italy; 4 Audiology Program, Department of Maternal, Child and Adult Medical and Surgical Sciences, University of Modena and Reggio Emilia, Modena, Italy; 5 Phoniatris and Audiology Unit, Department of Neuroscience DNS, University of Padova, Treviso, Italy; Universiti Malaya Fakulti Perubatan: University of Malaya Faculty of Medicine, MALAYSIA

## Abstract

**Purpose:**

Artificial Intelligence (AI) is increasingly being applied in the field of audiology, demonstrating potential to support screening, diagnosis, and rehabilitation in auditory and vestibular disorders. However, its effectiveness in guiding complex therapeutic decisions—such as those for idiopathic sudden sensorineural hearing loss (ISSNHL)–remains uncertain. This study aimed to compare treatment recommendations for ISSNHL made by medical doctors (MDs) with those proposed by an AI system (ChatGPT, GPT-4o model). The goal was to evaluate the concordance between human and AI decision-making, particularly regarding the administration of corticosteroids and adjunctive therapies, and to assess AI’s potential in clinical practice.

**Methods:**

This study is a retrospective observational analysis. Data from 86 patients diagnosed with ISSNHL were retrospectively analysed. Treatment decisions by MDs were compared with those generated by ChatGPT using anonymized patient datasets and a series of sequential prompts simulating multidisciplinary discussion. Agreement between AI and MDs was assessed using Cohen’s Kappa coefficient.

**Results:**

Overall, poor to fair agreement was observed between AI and clinician treatment decisions. ChatGPT did not recommend oral corticosteroids in 26 cases where MDs prescribed them (Kappa = 0.029) and recommended intravenous corticosteroids in 20 patients who were not treated with this approach by MDs (Kappa = 0.393). Discrepancies were also evident in recommendations for intratympanic steroids (Kappa = −0.044) and adjunctive therapies (Kappa = 0.035). These differences likely stem from the AI’s rigid adherence to generalized treatment protocols and limited contextual understanding.

**Conclusion:**

While ChatGPT (GPT-4o) shows promise in generating structured, protocol-driven suggestions, its clinical decision-making capabilities for ISSNHL remain inferior to those of experienced physicians. The AI’s lack of individualized reasoning and context sensitivity resulted in frequent discordance with physician-led care. Thus, AI should currently be viewed as a supportive tool in audiological practice, with its integration requiring careful oversight and further validation in real-world clinical environments.

## Introduction

Sudden sensorineural hearing loss (SSNHL) is defined as decrease in hearing threshold of 30 dB or more, affecting bone conduction in at least 3 consecutive frequencies, and occurring in a time frame within 72 hours [1]. Idiopathic sudden sensorineural hearing loss (ISSNHL) is defined as SSNHL with no identifiable cause despite thorough investigation. It is a relevant otologic emergency, accounting for approximately 1% of all sensorineural hearing loss cases, with an incidence between 8 and 15 cases per 100,000 individuals per year. However, determining the exact impact of various aetiologies on ISSNHL, remains challenging due to the frequent inability to ascertain the underlying cause of the condition and the presence of comorbidities [2]. As a result, identifying appropriate treatment options, and predicting functional prognosis currently remains difficult.

The recent development of artificial Intelligence (AI) technologies is currently revolutionizing many fields in medicine, allowing clinicians to implement novel precise diagnosing and prognostic tools, to support decision making [3]. In the field of audio-vestibular healthcare, the recent literature highlights a range of AI applications in audiology, from enhancing the accuracy of hearing assessments to automating the fitting of hearing aids and cochlear implants (CIs), as well as optimizing auditory rehabilitation processes [4–7]. During the last few years, the AI technologies have increasingly impacted the audiological diagnostic techniques [8–10]. It has been reported that machine learning (ML) can support diagnostic process, with performance comparable to that of clinicians, possibly allowing for the implementation of screening programs, particularly useful in rural and underserved communities [11]. From a predictive point of view, it has been reported that ML models could predict hearing levels, based on patient demographics, clinical factors, and subjective hearing status, thus identifying individuals who would benefit most from an audiological evaluation [12,13]. Moreover, adaptive AI models have shown promising results in predicting adult cochlear implant candidacy based on standard behavioural audiogram data [14]; although technical and regulatory challenges remain in routine clinical practice, studies have demonstrated the feasibility of audiological self-assessment and remote CI fitting with AI under the supervision of an audiologist, particularly among certain CI recipients [15,16].

Thus, according to the literature, AI application in various domains of audiology—including screening, diagnosis, rehabilitation, and follow-up of hearing loss—has being regarded with increasing interest, potentially allowing to (i) enhance the capabilities of audiologists, (ii) alleviate the burden of routine tasks, and (iii) provide personalized patient care [3]. Nevertheless, several open questions remain regarding medical AI applications both in general (data privacy, the size and quality of datasets for training algorithms, potential biases in AI models, and the need for rigorous validation before clinical implementation) [3], and in the specific setting of SSNHL. In fact, the lack of evidence about the use of AI-powered tools to predict SSNHL functional outcome still suggests caution in using them for counselling purpose, in this subset of patients, thus calling for further analysis to investigate the potential outcomes associated with the use of AI. The aim of this exploratory study was to compare the treatment recommendations made by medical doctors (MDs) with those proposed by an AI system, in a series of SSNHL cases. We look to evaluate and compare the different approaches used by MDs and AI, examining the similarities and differences in their treatment suggestions. Additionally, the study investigated and statistically analysed the AI’s proposed treatment plans, with reference to the individual medical conditions and histories of each patient.

## Materials and methods

### Patients

The study was conducted according to the principles of the Helsinki Declaration. Data were examined in agreement with Italian privacy and sensitive data laws. The study was approved by the Ethical Committee of the Province of Treviso, Italy (n° 1344/CE Marca, 25 May 2023). Participants provided their written informed consent to the use of their medical data for research purpose.

The study design was based on a retrospective multicentre series of patients admitted to the Phoniatrics and Audiology Unit of the University of Padova and the Audiology Unit of the University of Modena and Reggio Emilia. Data were accessed in a period between July 2023 and November 2023 and organized in an anonymized dataset.

Inclusion criteria comprised: (i) a diagnosis of ISSNHL, defined as a sensorineural hearing loss of ≥30 dB affecting at least three consecutive frequencies within a 72-hour period and treated at one of the two tertiary academic audiological centres; (ii) age ≥ 18 years; and (iii) availability of comprehensive clinical and audiological outcome data, including full medical history, pure-tone and speech audiometry, tympanometry, stapedial reflex testing, treatment details, and neuroimaging (temporal bone CT and brain MRI).

Exclusion criteria were: (i) hearing loss secondary to identifiable aetiologies such as vestibular schwannoma, endolymphatic hydrops, meningitis, trauma (including head trauma, temporal bone fracture, and acoustic trauma), barotrauma, or perilymphatic fistula; (ii) occupational noise exposure ≥80 dB; (iii) pre-existing known unilateral or bilateral hearing loss (excluding presbycusis) prior to the ISSNHL episode; (iv) disorders affecting the external or middle ear; (v) prior ear surgery; and (vi) lack of consent for data usage in research.

Collected data encompassed demographic information, presenting audio-vestibular symptoms (subjective hypoacusis, aural fullness, otalgia, tinnitus, flu-like symptoms, and vestibular signs), systemic comorbidities (e.g., diabetes, hypertension, vascular or autoimmune diseases), audiometric thresholds at presentation and post-treatment, and, when available, results of second-level diagnostic evaluations (e.g., auditory brainstem responses, CT imaging of the temporal bone, and/or MRI of the cerebellopontine angle).

Audio-vestibular symptoms and signs were operationally defined as follows: (i) subjective hypoacusis—self-perceived reduction in hearing acuity; (ii) fullness—a subjective sensation of aural pressure; (iii) vestibular symptoms including vertigo (illusory perception of motion) and disequilibrium (imbalance or unsteadiness, particularly during ambulation); (iv) spontaneous or positional nystagmus observed on clinical examination; (v) latero-deviation; and (vi) asymmetric vestibular response on caloric testing. The same physicians made the diagnostic and therapeutic decisions across patients in each centres. A unified database system was employed for data archiving across both centres.

The primary objective of this study was to determine the treatment indications for patients with ISSNHL from both artificial intelligence (AI) systems and trained physicians. Secondary, the study aimed to evaluate and compare the concordance between human and AI decision-making processes, with particular emphasis on the administration of corticosteroids and adjunctive therapies in clinical settings. This comparison sought to assess the potential future application of AI in clinical decision-making within audiology, specifically for patients presenting with ISSNHL.

### Diagnostic and therapeutic work-up

At diagnosis, each patient underwent an audiological study including (i) pure-tone audiometry on the 250–500–1000–2000–4000–8000 Hz frequencies by both air and bone conduction, (ii) speech audiometry, and (iii) tympanometry with stapedial reflex measurement. The pure-tone average (PTA) was estimated as the mean value of the 500–1000–2000–4000 Hz thresholds. From speech audiometry curves, the following thresholds were considered: (i) detection threshold, (ii) 50% speech discrimination score threshold (SDS50), and (iii) 100% speech discrimination score threshold.

Second-level tests included Auditory Brainstem Response (ABR), contrast-enhanced cerebellopontine angle MRI, temporal bone CT scan, and blood tests (white blood cell count [WBC], haemoglobin [Hb], platelets’ [PLT], neutrophils’, lymphocytes’, monocytes’ counts, C-reactive protein [CRP] and erythrocyte sedimentation rate [ESR]).

In all patients, treatment was initiated as soon as the baseline hearing status had been defined, so the time to diagnosis corresponded to the treatment delay. A systemic steroid therapy was administered to all patients, either orally with prednisone (0.5 to 1 mg/kg) or intravenously with methylprednisolone (0.5 to 1 mg/kg). When clinically indicated, steroid therapy was associated with other drugs, including multi-vitaminic complexes and, in the case of vertigo, betahistine. Second-line therapy included hyperbaric oxygen therapy (HBOT) (at least 20 sessions of 40 min, with an intervening 5-min air brake to prevent oxygen toxicity, under 100% O2 at a constant pressure of 2.2 atmospheres) and/or intratympanic steroid injections (a course of 3 intratympanic injections of 0.5–1 cc of dexamethasone 10 mg/mL, over 10 days).

For a detailed breakdown of the therapeutic strategies employed, see the Results section.

### ChatGPT query

#### Analysis tool.

The analysis of clinical data was conducted using exclusively the ChatGPT AI model, version GPT-4o (2023), developed by OpenAI. This tool was employed to analyse clinical data, formulate therapeutic hypotheses, and compare the obtained results with those published in the previous study [2].

ChatGPT was utilized through a series of sequential and specific prompts, simulating a discussion among various medical specialists, including an internist, an otolaryngologist with expertise in audiology, an emergency medicine physician, and an infectious disease specialist.

#### Input and prompts.

The analysis was conducted by creating a primary prompt, which was refined based on the AI’s responses, leading to the submission of over 50 variations of the same prompt. An accompanying spreadsheet with anonymized clinical details was provided, including age, sex, audiometric data, and medical history of patients diagnosed with sudden sensorineural hearing loss (SSNHL).

The final prompt, anonymized clinical data, and various requests to refine the AI’s responses and reassess the analysis were all submitted in a single new chat, allowing the model to maintain local context.

ChatGPT was asked to suggest appropriate treatments, discuss therapeutic options (including steroids, antivirals, and hyperbaric oxygen therapy), and evaluate potential protocol changes based on clinical outcomes.

#### AI model and session management.

The analysis of clinical data was conducted using exclusively the ChatGPT AI model, version GPT-4o (2023), developed by OpenAI. This tool was employed to analyse clinical data, formulate therapeutic hypotheses, and compare the obtained results with those published in the previous study [2].

We used ChatGPT (GPT‑4o) within a single conversation thread to maintain local context across iterative refinements of the prompt. No manual hyperparameters were set; the platform’s default values for temperature, top‑p, max tokens, and frequency/presence penalties were used.

#### Data inputs provided to the AI.

An anonymized spreadsheet was uploaded containing per‑patient demographics, comorbidities, timing (days from symptom onset), audiological measures (PTA at 0.5–4 kHz; frequency‑specific thresholds; stapedial reflexes), speech audiometry thresholds, symptom flags (e.g., tinnitus, aural fullness, vestibular symptoms), and hematological parameters (WBC, Hb, PLT, neutrophils, lymphocytes, monocytes, CRP, ESR). When fields were missing, the AI was instructed to base recommendations only on available data.

#### Prompt governance and standardization.

Prompting followed a controlled, template‑based approach. We designed a master prompt specifying: (i) clinical roles to simulate (internal medicine, ENT/audiology, emergency medicine, infectious diseases), (ii) decision objectives (therapy type, dosage, duration), (iii) constraints (use patient‑specific risk factors, audiological and hematological data; avoid unsupported assumptions), and (iv) the required output schema. Iterative variants were used only to add clarity on data columns and to enforce concise, conclusion‑first summaries. The final prompt template (S1 Appendix) was locked prior to generating the full set of patient‑level recommendations.

#### Output schema and coding rules.

For each patient, the AI produced a structured conclusion listing recommended therapies (oral corticosteroids; intravenous corticosteroids; intratympanic corticosteroids; adjunctive therapies such as multivitamins/betahistine; and HBOT), together with dosage and duration. We converted the narrative into five binary variables (recommended vs. not recommended) per category, using the following mapping: “recommend”, “indicated”, “initiate”, or explicit dosing → recommended; “consider only if”, “optional”, or conditional phrasing without clear initiation → not recommended; explicit negations → not recommended. Ambiguities were resolved by conservative coding (not recommended) unless an explicit initiation was present. The coders were blinded to physician decisions.

#### Reproducibility resources.

To facilitate replication, we provide the standardized prompt template (S1 Appendix) and the data dictionary of the spreadsheet columns within the prompt template. The dataset analyzed by the AI was the same dataset used to compute agreement statistics, sharing identical column names and patient identifiers.

#### Agreement analysis.

The agreement between the two evaluators was measured using Cohen’s Kappa coefficient. Specifically, agreement was assessed between medical doctors’ (MDs) and ChatGPT-generated treatment recommendations for each patient across predefined therapeutic categories, including: oral corticosteroids, intravenous corticosteroids, intratympanic corticosteroids, adjunctive pharmacological therapies (e.g., multivitamin complexes and betahistine), and HBOT. Each treatment option was coded as a binary variable (recommended vs. not recommended) for both evaluators, and Cohen’s Kappa was calculated separately for each therapeutic category. Cohen’s Kappa values were interpreted according to the benchmarks proposed by Landis and Koch [17]: values <0 indicating poor agreement, 0.00–0.20 slight, 0.21–0.40 fair, 0.41–0.60 moderate, 0.61–0.80 substantial, and >0.80 almost perfect agreement. For each Cohen’s Kappa coefficient, standard errors were obtained from SPSS output, and 95% confidence intervals were calculated using the normal approximation (κ ± 1.96 × SE). Cohen’s Kappa values were interpreted according to the benchmarks proposed by Landis and Koch [17].

Given the heterogeneous distribution of audiometric thresholds, continuous variables were described using both parametric (mean ± standard deviation) and non-parametric measures (median, interquartile range, and quartiles). Pure-tone average (PTA) values were reported exclusively for descriptive purposes to characterize the clinical sample, and no parametric inferential analyses involving PTA were performed.

All agreement analyses were conducted with an exploratory aim to describe concordance across distinct therapeutic categories rather than to formally test a single family of hypotheses. Accordingly, no correction for multiple comparisons was applied.

## Results

### General clinical features and medical doctor indications for treatment

According to the inclusion/exclusion criteria, data from 86 patients (38 females, 48 males; median age: 58.00 years [IQR: 47.00–69.00]) were analysed. The distribution of demographics, clinical features, and comorbidities, as well as hearing thresholds at diagnosis, has been summarized in Table 1.

**Table 1:** Demographics, clinical features, comorbidities, and hearing thresholds at diagnosis.

Sample size	86 (38 M, 48 F)
Age (mean±SD), in years	57.05 ± 15.8 Range 21–88
PTA (mean±SD), in dB	57.05 ± 26.5 Range 22–130
Comorbidities (N)	Unknown (5) Absent (32) Present (49)
Audio-vestibular symptoms (N)	Unknown (2) Absent (0) Present (84)

PTA: pure tone averages

Based on medical doctor indications, 83 out of 86 patients received a first-line oral steroid treatment, starting with a median dose of 30.00 mg of Prednisone (IQR: 25.00–50.00 mg). Ten patients received intravenous (IV) steroids (two as upfront treatments, eight as a second-line therapy after oral steroid administration), starting with a median methylprednisolone dose of 60.00 mg (IQR: 60.00–60.00 mg). Intratympanic steroid injection was administered as a second-line therapy in two cases after an upfront oral treatment. Forty-five patients also received concomitant therapy with other drugs (including multi-vitamin complexes and, in cases of vertigo, betahistine). In 21 cases, HBOT was performed as a second-line treatment.

In addition to mean and standard deviation, non-parametric descriptive statistics were reported for PTA. The median PTA was 48.1 dB, with an interquartile range (IQR) of 36.3–74.1 dB. The first and third quartiles (Q1–Q3) were 36.3 dB and 74.1 dB, respectively.

### ChatGPT indication for treatment

A marked discrepancy was observed between the treatment recommendations of the AI model and those of medical doctors (MDs) with regard to the prescription of oral corticosteroids. Specifically, the AI did not recommend oral steroid therapy in 26 cases, whereas physicians prescribed oral steroids in 25 of these same cases. The calculated Cohen’s Kappa coefficient for this comparison was 0.029, indicating a poor level of agreement.

When evaluating the prescription of IV corticosteroids, a moderate level of discordance was identified. The AI recommended IV steroid administration in 20 patients who were not managed with this approach by MDs. The corresponding Cohen’s Kappa (Table 2) value was 0.393, denoting fair agreement. The principal source of disagreement appeared to stem from the AI’s tendency to favour IV administration over oral routes, possibly based on prognostic factors such as severity of hearing loss or presence of comorbid conditions that were interpreted differently by the AI model compared to human clinical reasoning.

**Table 2:** Agreement between therapies auggested by AI vs ENT (ear nose throat) Specialists.

	Suggested therapies (number of patients)		Mesure of Agreement (Kappa)				95% C.I. (confidence intervals)
	ENT Specialist	AI	Cohen’s Kappa	Asymptotic Standard Error	Approximate T-value	p-value	
Oral Corticosteroid	83	60	.029	.055	.603	.547	−0.08 to 0.14
Intravenous corticosteroid	10	30	.393	.094	4558	<.001	0.21 to 0.58
Intratympanic corticosteroid	2	16	−.044	.028	−.689	.491	−0.10 to 0.01
Additional therapies	45	25	.035	.096	.365	.715	−0.15 to 0.22
Hyperbaric oxygen therapy	15	20	.121	.125	1.02	.308	−0.12 to 0.37

The analysis of intratympanic steroid therapy revealed substantial inconsistency between AI-generated and clinician-driven decisions. The AI did not recommend intratympanic corticosteroid injections in 67 out of 83 cases. The Cohen’s Kappa value was −0.044, indicating worse-than-chance agreement, suggestive of a negative correlation between AI and physician treatment patterns.

With respect to the recommendation of adjunctive therapies—such as multivitamin complexes or anti-vertiginous agents like betahistine—there was again poor concordance. The AI declined to propose additional therapies in 31 patients for whom MDs opted to provide such interventions. Conversely, in 11 cases, the AI suggested additional treatments not prescribed by the attending clinicians. The resulting Cohen’s Kappa coefficient of 0.035 confirms poor agreement. Regarding hyperbaric oxygen therapy (HBOT), the AI suggested its use in 20 patients, compared to 15 cases where it was administered by MDs. The corresponding Cohen’s Kappa was 0.121, indicating slight agreement.

## Discussion

This study highlighted the potential utility of ChatGPT (GPT-4o) as a supportive tool in clinical decision-making for ISSNHL, exploring the concordance between treatment decisions made by medical doctors (MDs) and those generated by the AI system. The choice of ChatGPT (GPT-4o) as the AI model for this study was due to its natural language framework, widespread adoption, and versatility in handling complex clinical narratives, representing a clinically relevant benchmark for evaluating the potential role of generative AI in medical decision-making [3–5]. Moreover, the possibility to simulate multidisciplinary clinical reasoning through sequential prompting made it particularly suitable for exploratory comparison with physician-led treatment decisions [4,5].

Overall, in this exploratory study, the results revealed generally poor to fair agreement, with the most notable discrepancies arising in the administration of oral and intratympanic steroids and additional therapies. These findings align with the broader landscape of AI in audiological care as reviewed by Frosolini et al. [4], who underscored that although AI models show substantial promise in diagnostic and predictive domains, clinical implementation—especially regarding personalized treatment decisions—remains limited by interpretability, context sensitivity, and the lack of validation against real-world outcomes. In our study, the Cohen’s Kappa values ranged from −0.044 to 0.393, highlighting that AI decisions often diverged from human clinical judgment, particularly when therapeutic nuances required consideration of comorbidities, clinical subtleties, or individualized reasoning.

This observation is consistent with the findings of other studies exploring the role of AI in different fields of audiology. For instance, Gathman et al. [12] and Wang et al. [13] demonstrated that machine learning (ML) models could predict pure tone averages (PTA) from demographics and subjective hearing complaints, which may be valuable for screening purpose, but not directly translatable to complex treatment decisions, like those for ISSNHL. Similarly, Crowson et al. [18] showed that deep learning could effectively interpret audiograms, and Heisey et al. [10] highlighted how dynamically masked audiograms could reduce testing time without compromising accuracy. Yet, these tools still function in a supportive, not substitutive, role.

The therapeutic management of ISSNHL is highly dependent on timing, patient-specific risk factors, comorbid conditions, and second-level diagnostics—all elements that were simplified or partially absent in the AI-based evaluation. The fact that AI frequently avoided recommending oral steroids (Kappa = 0.029), while MDs consistently did, as a first-line treatment, reflects a possible tendency towards over-conservatism, risk aversion, and/or strict observance of guideline indications by the algorithm, possibly related to limited contextual understanding or data sensitivity thresholds embedded in the model [19].

Conversely, the AI suggested intravenous steroid use in 20 patients where MDs did not (Kappa = 0.393), and this might indicate AI’s tendency to apply aggressive second-line treatments earlier, potentially extrapolated from generalized protocols without patient-specific clinical nuances. Similar trends were observed by McKearney et al. [7], where ML models outperformed traditional detection methods for auditory brainstem responses (ABR) using simulated data. Thus, translation to clinical decision-making seem to still require human oversight, particularly in specific conditions and waiting for AI databases implementation and improvements of its functioning [20].

Furthermore, Jin et al. [10] showed that AI could equal audiologists in detecting middle ear diseases via tympanograms, including those performed by laypersons. While encouraging, these findings emphasize AI’s strength in pattern recognition tasks, whereas in this study, decision-making based on non-patterned, contextualized judgment was less reliable.

The scoping review by Frosolini et al. [3] echoes these conclusions, noting that AI shows excellent potential for enhancing diagnostic accuracy and supporting audiological assessments, but also emphasizes critical challenges such as data heterogeneity, algorithmic bias, and the insufficiency of AI validation in dynamic clinical environments. These limitations are particularly salient in ISSNHL, where diagnosis is typically a diagnosis of exclusion and treatment often involves managing uncertainties.

Moreover, our study’s approach of simulating a multidisciplinary medical discussion within ChatGPT aligns with efforts to make AI outputs more robust. However, as also recognized by Meeuws et al. [15] and Wathour et al. [16] in the context of remote cochlear implant (CI) fitting, AI tools still require human supervision, particularly when adapting protocols or evaluating complex patient profiles.

Beyond overall agreement metrics, the observed discrepancies between clinicians’ and AI-generated treatment recommendations may be partially explained by differences in how specific clinical parameters are weighted during decision-making. In particular, clinicians are likely to integrate audiometric severity, comorbid conditions, vestibular symptoms, and the timing of presentation within a broader contextual and experiential framework. Conversely, the AI system appeared to rely more heavily on protocol-driven interpretations of hearing loss severity and generalized indications for second-line therapies, potentially favouring more aggressive or standardized approaches in selected cases.

Importantly, this study was not designed to formally test the influence of individual clinical variables on treatment decisions, and no causal inferences can be drawn. Rather, this exploratory analysis aims to contextualize the observed discordance by highlighting plausible clinical factors that may differentially shape human and AI-driven therapeutic preferences.

Overall, our study has some strengths, mainly lying in its ability to systematically analyse large datasets, simulate multidisciplinary discussions, and generate protocol-based treatment suggestions. The AI system demonstrated consistency in applying generalized therapeutic criteria and offered alternative perspectives, which may be useful in standardizing care or identifying areas for further clinical review. However, some limitations emerged: ChatGPT does not have clinical experience and relies exclusively on text-based input. It cannot access diagnostic images or detailed patient data, and its responses are based on information available up to 2023 (see materials and methods section) and may not reflect the latest evidence or guidelines. Moreover, the model often adhered rigidly to standard protocols, displaying limited capacity for individualized reasoning or inferential flexibility. These factors contributed to poor-to-fair agreement with physicians’ decisions, emphasizing the necessity for human oversight when integrating AI into clinical practice.

## Conclusion

This study suggested that, while AI models such as ChatGPT (GPT-4o) could offer structured and protocol-driven treatment suggestions for ISSNHL, their clinical decision-making remains limited when compared to that of experienced physicians. The observed poor to fair agreement—particularly in nuanced therapeutic areas like steroid administration and adjunctive treatments—highlighted AI’s current role as a supportive rather than substitutive tool. Despite its potential for data synthesis and standardization, AI appeared to lack the contextual understanding and individualized reasoning essential for managing complex clinical scenarios. Therefore, its integration into audiological care should remain under close human supervision, with further research needed to enhance model adaptability, validation, and clinical safety.

## Supporting information

S1 Appendix Final prompt template employed to generate the full set of patient‑level recommendations. (PDF)
