# Supplementary material for: Human versus AI in audiological practice: A comparative evaluation of ChatGPT and physician treatment decisions in idiopathic sudden sensorineural hearing loss
Source: PLoS One. 2026 Jun 1;21(6):e0350549. doi: 10.1371/journal.pone.0350549 (PMC13225402; doi:10.1371/journal.pone.0350549)
Supplement: S1 Appendix — (PDF) [file pone.0350549.s001.pdf]

Appendices

Appendix A - MASTER PROMPT TEMPLATE

Roles to Simulate:

- Internal Medicine physician
- ENT/Audiology specialist
- Emergency Medicine physician
- Infectious Disease specialist

Objective:

For each patient (row) in the uploaded spreadsheet, produce a concise, conclusion-first recommendation that includes: (1) therapy type(s) among oral corticosteroids, intravenous corticosteroids, intratympanic corticosteroids, HBOT, antivirals, or other; (2) dosage and duration; (3) whether HBOT is useful (timing and number of cycles); (4) whether antivirals are needed (linked to symptoms and haematology).

Data Dictionary (columns):

A: patient_code

B: center_code (if present)

C: sex (M/F)

D: age_years

E: days_since_onset

F: hypertension (0/1)

G: diabetes (0/1)

H: vascular_disease (0/1)

I: dyslipidemia (0/1)

J: renal_insufficiency (0/1)

K: ototoxic_drugs (0/1)

L: autoimmune/rheumatologic (0/1)

M: PTA_affected (dB)

N: threshold_250Hz (dB)

O: threshold_500Hz (dB)

P: threshold_1000Hz (dB)

Q: threshold_2000Hz (dB)

R: threshold_4000Hz (dB)

S: threshold_8000Hz (dB)

T: stapedial_reflex_present (0/1)

U: stapedial_500Hz (dB)

V: stapedial_1000Hz (dB)

W: stapedial_2000Hz (dB)

X: stapedial_4000Hz (dB)

Y: speech_detection (dB)

Z: speech_SDS50 (dB)

AA: speech_100pct (dB)

AB: PTA_contralateral (dB)

AC: subjective_hypoacusis (0/1)

AD: aural_fullness (0/1)

AE: tinnitus (0/1)

AF: vestibular_symptoms (0/1)

AG: otalgia (0/1)

AH: flu_like_symptoms (0/1)

AI: WBC

AJ: Hb

AK: PLT

AL: neutrophils

AM: lymphocytes

AN: monocytes

AO: CRP

AP: ESR

Instructions:

- Use only available fields; if a field is missing, do not infer it.

- Integrate audiological severity (PTA, reflexes), comorbidities, timing, and hematology.

- Prefer conclusion-first style; keep the multidisciplinary discussion minimal.

- Output must follow the schema below.

Required Output Schema (per patient):

Patient {patient_code} — Center {center_code}:

Key data: age {age_years}, days_since_onset {days_since_onset}, PTA {PTA_affected} dB, stapedial_reflex {stapedial_reflex_present}, comorbidities [list].

Conclusion: [Therapy types]

Dosage/Duration: [details]

HBOT: [useful/not; timing; cycles]

Antivirals: [needed/not; rationale linked to hematology/symptoms]

Coding Guidance:

Map recommendations to binary variables for each category (oral, IV, intratympanic, adjunctive, HBOT). Only explicit initiation counts as 'recommended'. Conditional or equivocal language counts as 'not recommended'.

Model Parameters:

Use platform defaults for temperature, top-p, max tokens, and frequency/presence penalties.
